# Burnout in trauma and orthopaedics: a cross-sectional study of surgeons from across the United Kingdom

**DOI:** 10.1308/rcsann.2022.0165

**Published:** 2023-03-16

**Authors:** SK Nayar, F Acquaah, B Kayani, K Vemulapalli

**Affiliations:** ^1^ Great Ormond Street Hospital for Children NHS Foundation Trust, UK; ^2^ Barts Health NHS Trust, UK; ^3^ University College London Hospitals NHS Foundation Trust, UK; ^4^ Barking, Havering and Redbridge University Hospitals NHS Trust, UK

**Keywords:** Burnout, Disengagement, Exhaustion, Orthopaedic, United Kingdom

## Abstract

**Introduction:**

Improving psychological wellbeing in healthcare professionals has demonstrable links with improvements in system-performance and patient outcomes. The aims of this study were to determine the prevalence of burnout among orthopaedic surgeons within the United Kingdom (UK) and to identify any influencing factors.

**Methods:**

This prospective, cross-sectional nationwide study used the Oldenburg Burnout Inventory to assess burnout among orthopaedic registrars, middle grades and consultants from across the UK. In total, 369 participants completed an anonymised online survey. Reasons for burnout were assessed using a list of 20 stressors followed by a white-space box for elaboration.

**Results:**

Respondents included 204 (55.3%) consultants, 100 (27.1%) registrars and 65 (17.6%) who were an associate specialist, trust grade or fellow. Some 252 (68.3%) participants experienced moderate-level burnout and 81 (22.0%) high-level burnout. There was no significant association between burnout and grade (*p* = 0.283), gender (*p* = 0.433), marital status (*p* = 0.932), years since graduation (*p* = 0.397), overseas training (*p* = 0.642), hours worked (*p* = 0.601), region (*p* = 0.699) or hospital type (*p* = 0.813). A high level of disengagement and exhaustion were identified, with the latter being a greater contributory factor. The most common reasons for burnout were insufficient staff numbers, an overload of administrative work and limited time to see patients.

**Conclusion:**

There was a moderate to high level of burnout among orthopaedic surgeons within the UK. Clinical and management teams should focus on improving staff numbers, reducing administrative work and increasing clinic consultation times to limit any further disengagement and exhaustion from surgeons. Reducing burnout may help to improve mental wellbeing, work satisfaction and workforce productivity.

## Introduction

In recent years, there has been a greater emphasis on recognising the psychological wellbeing of doctors. Among British doctors in general, morale has been found to be at an all-time low, which has been exacerbated by the Covid-19 pandemic. This is evidenced by the ever-reducing numbers of doctors going from foundation training into specialty training, with just 37.7% of Foundation Year 2 doctors applying to continue training in 2018, down from 71.3% in 2011.^1^ An important issue contributing to this is the rise in burnout.^2^

Burnout is defined in the 11th revision of the International Classification of Diseases (ICD-11) as: ‘a syndrome conceptualised as resulting from chronic workplace stress that has not been successfully managed. It is characterised by three dimensions: feelings of energy depletion or exhaustion; increased mental distance from one’s job, or feelings of negativism or cynicism related to one's job; and reduced professional efficacy. Burnout refers specifically to phenomena in the occupational context and should not be applied to describe experiences in other areas of life’.^3^

Several studies have investigated physician burnout, with a recent cross-sectional study of 1,673 British doctors across multiple specialties reporting one-third of doctors suffer from burnout.^2^ Reasons for this appear to be multifactorial.^4-7^ Common contributing factors include excessive workloads, lack of autonomy, external pressures and dysfunctional workplaces.

Improving psychological wellbeing in healthcare professionals has demonstrable links with improvements in system-performance and patient outcomes.^8,9^ It is therefore vital that burnout and the reasons for it are identified and changes are implemented to improve welfare. This is particularly pertinent in the British National Health Service (NHS), which continues to be pushed to new limits year on year.

The primary aim of this study was to determine the prevalence of burnout among senior orthopaedic surgeons (registrars, middle grades and consultants) within the UK. Secondary aims were to identify factors that influence burnout and establish any precipitating stressors for burnout in orthopaedic surgeons.

## Methods

A prospective, cross-sectional nationwide study was carried out. The eligibility criteria were restricted to doctors practising in the UK NHS including specialist registrars, middle grades and consultants in orthopaedic surgery. Participants were asked to complete an anonymised online survey via Google Forms. The online survey was disseminated via email invitation and social media. Data collection occurred between 4 January and 31 March 2022. The survey consisted of three sections: demographic information, a burnout inventory and reasons for burnout (Tables 1–3, respectively).

**Table 1:** Demographic information

Question	Multichoice answer
Current grade	Junior registrar (ST3–5)
	Senior registrar (ST6–8)
	Associate specialist
	Trust grade
	Consultant
	Other – specify
Are you a clinical lead/director or equivalent?	Yes
		No
	Years since graduation	0–5
			5–10
			10–20
			20+
		Are you a UK graduate?	Yes
				No
			Gender	Male
					Female
					Prefer not to say
				Marital status	Married
						Cohabitating with significant other
						Single
						Divorced
						Prefer not to say
					Hours worked	Full time
							Part time – specify %
						Region in which you are working	
						Type of hospital in which you are working	District general hospital
								Major trauma centre
								Elective tertiary centre
								Other – specify

**Table 3:** Reasons for burnout

1	Work overload
2	Limited time to see patients
3	Lack/non-functionality of diagnostic tools
4	Fear of incorrect/suboptimal decision making
5	Fear of intra-/postoperative complications
6	Overload of administrative work
7	Hours at work
8	Insufficient staff numbers
9	Communication with patients and/or family members
10	Finance/personal income
11	Lack of respect from other staff members
12	Negative public attitude towards profession
13	Mismatch between expectations and reality of the job
14	Unrealistic expectations of patients
15	Lack of sleep
16	Lack of exercise/meditation/mind rest
17	Fear of job loss
18	Possibility of legal claims and litigation
19	Competition in private practice
20	Friends/family/loved ones
21	Please elaborate on any areas that cause you to feel stressed or burned out

Questions 1–20 graded as ‘not at all’, ‘a little’, ‘moderately’ or ‘a lot’

Burnout was assessed using the Oldenburg Burnout Inventory (OLBI; Table 2). This is a validated tool to assess burnout.^10-14^ It is composed of 16 items and subcategorises burnout into two key domains, namely exhaustion and disengagement. These domains are each represented by eight items, with four being positively worded and four negatively worded, arranged in a mixed pattern for psychometric balancing.^10^ Items 2, 4, 5, 8, 10, 12, 14 and 16 explore exhaustion, whereas 1, 3, 6, 7, 9, 11, 13 and 15 explore disengagement. Each item is scored on a Likert-type scale as: ‘strongly agree – 1’, ‘agree – 2’, ‘disagree – 3’ and ‘strongly disagree – 4’. Reverse scoring is applied to the items marked with an ‘R’ on the tool (e.g. strongly agree – 4 and strongly disagree – 1). A higher score represents higher exhaustion and disengagement, and hence higher levels of burnout. An overall score < 30 was considered low burnout, 30–45 moderate burnout and > 45 high burnout.^15^ Mean cut-off scores > 2.1 and > 2.25 were considered as high levels of disengagement and exhaustion, respectively.^16,17^

**Table 2:** Oldenburg Burnout Inventory

1	I always find new and interesting aspects in my work
2	There are days when I feel tired before I arrive at work
3	It happens more and more often that I talk about my work in a negative way
4	After work, I tend to need more time than in the past in order to relax and feel better
5	I can tolerate the pressure of my work very well
6	Lately, I tend to think less at work and do my job almost mechanically
7	I find my work to be a positive challenge
8	During my work, I often feel emotionally drained
9	Over time, one can become disconnected from this type of work
10	After working, I have enough energy for my leisure activities
11	Sometimes I feel sickened by my work tasks
12	After my work, I usually feel worn out and weary
13	This is the only type of work that I can imagine myself doing
14	Usually, I can manage the amount of my work well
15	I feel more and more engaged in my work
16	When I work, I usually feel energized

All questions graded as ‘strongly agree’, ‘agree’, ‘disagree’ or ‘strongly disagree’

Reasons for burnout were assessed using a list of 20 stressors. This list was formulated from previously reported reasons for burnout among physicians and surgeons, as well as other anecdotal reasons for burnout.^18^ Each item was scored on a Likert-type scale as ‘not at all – 0’, ‘a little – 1’, ‘moderately – 2’ or ‘a lot – 3’. A white-space box with no word or character limit was included at the end of the survey asking participants to elaborate on any areas that cause them to feel stressed or burned out.

### Statistical analysis

Statistical analysis was performed using GraphPad Prism 7 (GraphPad Software, San Diego, US). Unpaired Student’s *t*-test was used for comparison between two groups and one-way analysis of variance for multiple groups. A *p-*value < 0.05 was considered statistically significant.

## Results

### Demographic information

In total there were 369 respondents, of whom 204 (55.3%) were consultants, 100 (27.1%) were registrars and the remaining 65 (17.6%) were an associate specialist, trust grade or fellow. Some 300 (81.3%) respondents were male and 65 (17.6%) were female. Sixty-seven (17.3%) were clinical leads or equivalent. A full breakdown of demographic information is provided in Table 4.

**Table 4:** Overall levels of burnout according to demographic parameter

Demographic parameter	No. of respondents (%)	OLBI score, mean ± SD	*p*-value
Current grade			0.283
Junior registrar (ST3–5)	52 (14.1)	40.9 ± 6.9	
Senior registrar (ST6–8)	48 (13)	40.6 ± 6.9	
Associate specialist	7 (1.9)	36.9 ± 7.1	
Trust grade	34 (9.2)	41.9 ± 6.4	
Fellow	21 (5.7)	37.8 ± 7.4	
Consultant	204 (55.3)	39.3 ± 8.3	
Other	3 (0.8)	41.3 ± 8.1	
Clinical lead/director or equivalent?			0.196
Yes	67 (18.2)	38.7 ± 8	
No	302 (81.8)	40.1 ± 7.7	
Years since graduation			0.397
0–5	18 (4.9)	39.7 ± 9	
6–10	82 (22.2)	41.1 ± 7.1	
11–20	114 (30.9)	39.7 ± 7.3	
> 20	155 (42)	39.3 ± 8.2	
UK graduate			0.642
Yes	247 (66.9)	40.0 ± 7.8	
No	122 (33.1)	39.6 ± 7.7	
Gender			0.433
Male	300 (81.3)	39.6 ± 7.8	
Female	65 (17.6)	40.8 ± 7.4	
Prefer not to say	4 (1.1)	42.3 ± 11.6	
Marital status			0.932
Married	274 (74.3)	39.7 ± 7.9	
Cohabitating with significant other	34 (9.2)	40.7 ± 7.6	
Single	39 (10.6)	40.2 ± 6.6	
Divorced	13 (3.5)	39.1 ± 7.3	
Prefer not to say	9 (2.4)	40.8 ± 8.7	
Hours worked			0.601
Full time	352 (95.4)	39.9 ± 7.7	
Part time	17 (4.6)	38.9 ± 7.4	
Region			0.699
East Midlands	12 (3.3)	40.8 ± 6.7	
East of England	37 (10)	41.6 ± 8	
North East	19 (5.1)	38.4 ± 7.7	
North West	47 (12.7)	40.6 ± 6	
Scotland	11 (3)	41.6 ± 9.5	
South West (Peninsula)	4 (1.1)	31.0 ± 10.2	
South West (Severn)	5 (1.4)	38.4 ± 10.6	
London	135 (36.6)	39.4 ± 7.7	
Kent, Surrey and Sussex	7 (1.9)	42.4 ± 8.8	
Thames Valley	8 (2.2)	37.7 ± 6.2	
Wessex	11 (3)	40.5 ± 9.6	
West Midlands	16 (4.3)	40.9 ± 8.5	
Yorkshire and the Humber	39 (10.6)	39.2 ± 6.9	
Wales	13 (3.5)	38.8 ± 9.9	
Norther Ireland	19 (5.1)	41.5 ± 14.8	
Other	3 (0.8)	42.7 ± 3.8	
Type of hospital			0.813
District general hospital	197 (53.4)	40.1 ± 7.6	
Major trauma centre	123 (33.3)	39.9 ± 8	
Elective tertiary centre	40 (10.8)	38.8 ± 7.7	
Other	9 (2.4)	39.6 ± 6.8	

OLBI = Oldenburg Burnout Inventory.

OLBI score summary: < 30 = low burnout, 30–45 = moderate burnout, >45 = high burnout

### Burnout

**Figure 1:**
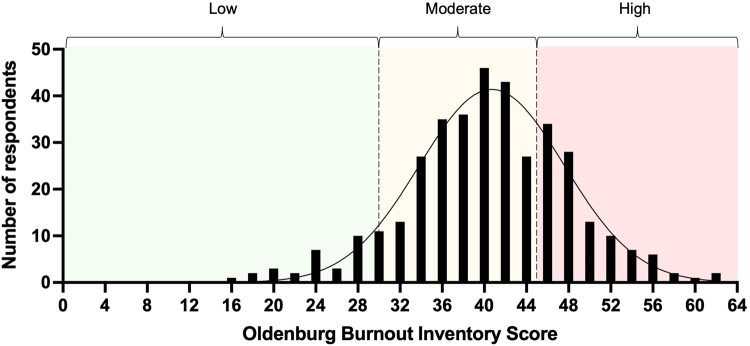
Frequency distribution of Oldenburg Burnout Inventory scores

The overall level of burnout was considered moderate among all respondents with a mean score of 39.8 on the OLBI (range 16 to 61). Thirty-six respondents (9.8%) reported low burnout, 252 (68.3%) moderate burnout and 81 (22.0%) suffered high burnout. The distribution of OLBI scores is illustrated in Figure 1.

There was no statistically significant association between amount of burnout and any of the demographic criteria outlined in Table 1. A non-significant trend towards lower levels of burnout were found in those based in the South West (Peninsula) area (mean OLBI score 31, *n* = 4) (Table 4).

**Figure 2:**
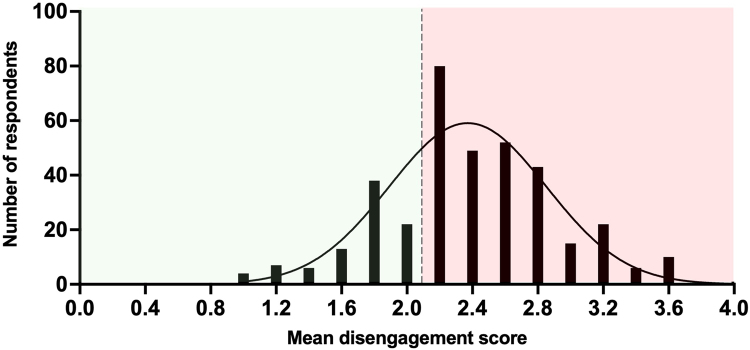
Frequency distribution of mean disengagement scores

**Figure 3:**
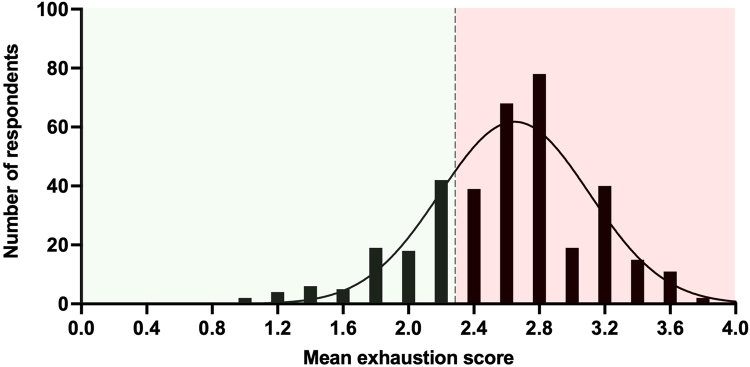
Frequency distribution of mean exhaustion scores

Mean disengagement and exhaustion scores ranged from 1 to 4 with a mean disengagement score of 2.39 and mean exhaustion score of 2.6, indicating high levels of both (Figures 2 and 3, respectively). There were higher levels of exhaustion than disengagement among respondents.

### Reasons for burnout

**Figure 4:**
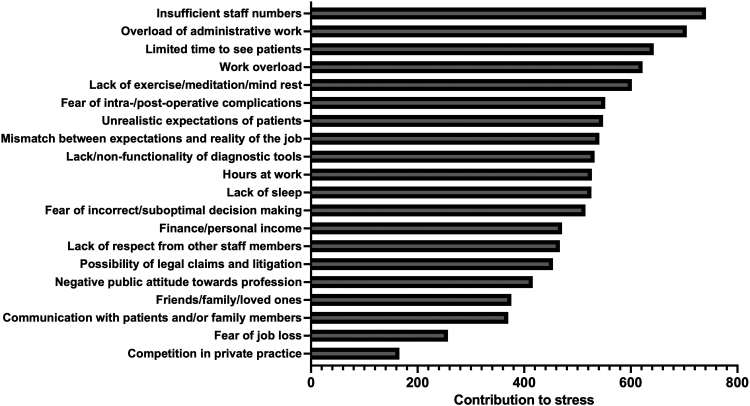
Relative contribution of individual stressors towards burnout

The most common reason for burnout was insufficient staff numbers, with 142 respondents (38.5%) reporting this as causing a high level of stress and 121 (32.8%) a moderate level of stress. This was followed by an overload of administrative work with 127 (34.4%) reporting this as causing a high level of stress and 118 (32.0%) a moderate amount of stress. A full breakdown of the relative contribution of each stressor towards burnout is illustrated in Figure 4.

**Figure 5:**
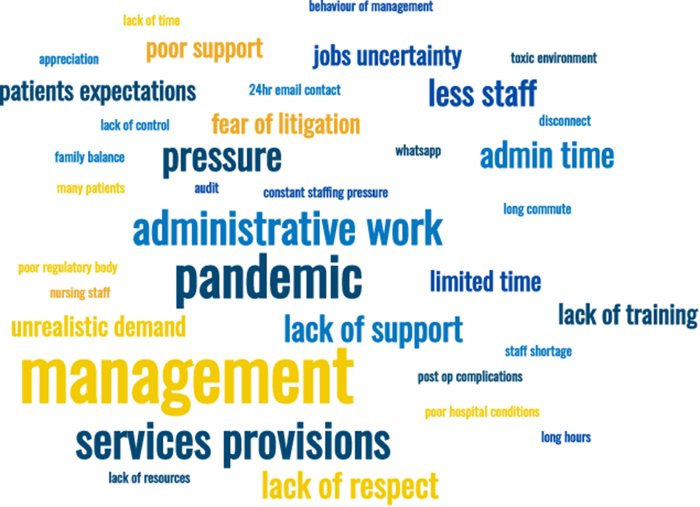
Word cloud of qualitative data on causes of burnout

In total, 113 participants provided additional written responses regarding contributing factors to their burnout. Key themes that emerged from these were understaffing, rising demands and expectations, pressures of the job, disproportionate service provision with a lack of training opportunities, the impact of the Covid-19 pandemic, the burden of administrative work, bullying and harassment, work–life balance, lack of certainty regarding location in training or availability of consultant jobs, perceived incompetence of management or administrative staff, insufficient support, working hours, poor IT systems, and lack of appreciation. Figure 5 illustrates a word cloud of key themes that emerged from answers to this question.

## Discussion

This study demonstrates burnout among orthopaedic surgery specialist registrars, middle grades, and consultants in the UK, with 68.3% experiencing moderate levels of burnout and 22.1% high levels of burnout. This is irrespective of grade, gender, marital status, years since graduation, overseas training, hours worked, region or hospital type. A high level of both disengagement and exhaustion were identified, with the latter being a greater contributory factor towards burnout.

Reasons for burnout appear to be multifactorial. In this study, the greatest contributory factors were insufficient staff numbers, overload of administrative work and limited time to see patients. All of these can be considered modifiable work-related factors and thus amenable to change.

This study supports findings from a recent survey published by the British Orthopaedic Association of 1,298 respondents using the Copenhagen Burnout Inventory, which found 40% of respondents to suffer burnout and a further 50% be at risk of burnout.^19^ This is in keeping with our study with 90.4% of respondents experiencing moderate to high burnout. Differences in the British Orthopaedic Association survey were higher rates of burnout in trainees and females, as well as in ethnic and LGTBQ+ groups, the latter two of which were not assessed in our study. Key stressors identified were issues with management and time pressures, which corroborate with our findings.

When comparing our findings with studies in other countries looking at burnout among orthopaedic surgeons, burnout rates vary considerably from 28% to 81.5% using validated and comparable burnout inventories (Oldenburg and Maslach) (Table 5).^18, 20-27^ Reasons for this variation can be explained by differences in the structure of healthcare delivery, working hours, working conditions and cultural differences. Lowest rates of burnout were found among orthopaedic trainees in the Netherlands.^22^ In the USA, burnout tended to be more linked to work hours, sleep deprivation and poor work–life balance, which were relatively less problematic in our cohort.^18^ This is likely related to the difference in work hours between the UK and USA, with working-time regulations restricting the number of hours trainees work in the UK. By contrast, work limitations such as staff numbers, administrative work and limited time to see patients played a larger role in our study, which may reflect the difference in the delivery of healthcare in these countries (nationalised vs privatised healthcare). Higher levels of burnout in our study may also reflect the impact of the Covid-19 pandemic, as evidenced by the written responses in our survey.

**Table 5:** Summary of burnout studies specific to orthopaedic surgeons

Study	Country	Cohort	Burnout
Nayar *et al* (2022, Current study)	UK	204 consultants 100 registrars 65 associate specialists/trust grades/fellows	Moderate burnout in 68% High burnout in 22%
Caesar *et al* (2021)^19^	UK	1,298 British Orthopaedic Association members	Burnout in 40% At risk of burnout in 50%
Zheng *et al* (2017)^21^	China	202 adult arthroplasty surgeons	Burnout in 81.5%
Van Vendeloo *et al* (2014)^22^	Netherlands	105 residents	Burnout in 28%
Arora *et al* (2014)^23^	Australia	51 residents	Burnout in 53%
Balch *et al* (2011)^24^	USA	155 surgeons	Burnout in 32%
Sargent *et al* (2009)^18^	USA	384 residents 264 faculty	High burnout in 56% of residents and 28% of faculty
Saleh *et al* (2009)^25^	USA	110 clinical leads	Burnout in 36%
Lesić *et al* (2009)^26^	Serbia	30 surgeons	Burnout in 40%
Sadat-Ali *et al* (2005)^27^	Saudi Arabia	69 surgeons	Burnout in 59.4%

Tackling burnout among orthopaedic surgeons, and surgeons in general, in the authors’ opinions is a public safety issue of pressing concern. Strategies to tackle burnout often focus on the individual, which in itself further potentiates stress because it puts even more responsibility on the already overworked surgeon and overlooks the root causes of burnout that are often at a system-wide level. In our study the factors that orthopaedic surgeons felt had the biggest influence on burnout were ones that arguably require an organisation/system-wide approach to address.

In this study, insufficient staff numbers had the largest contribution to burnout in orthopaedic surgeons. There are multiple factors that contribute to this. At the junior level, the number of doctors progressing directly into specialist training reduces year on year.^28^ This is largely due to the prospect of better work–life balance and higher income through alternative routes such as locum work, working in other countries such as Australia, or leaving the healthcare profession altogether.^1^ This is particularly pertinent in the current economic climate with the rising cost of living. At the senior end, there is an increasing tendency to retire early, potentiated by issues with the NHS pension scheme and taxation.^29^ This reducing workforce creates increasing pressures on those that remain in the system, further enhancing rates of burnout, leading to even more surgeons leaving the profession, hence creating a positive feedback loop. Further compounding this are staff shortages from across the multidisciplinary team, in particular the staffing crisis among nurses.^30^

Strategies to mitigate this include employing more staff, new ways of working, reducing workload and improving staff retention. In practice, this can be achieved by an increased financial settlement from the government to offset a decade of austerity and cuts to spending on the NHS.^31^ Investing in the existing workforce and a focus on improving wellbeing would improve staff retention and consequently have a significant cost benefit to the NHS.^32^ One focus should be improving working conditions, such as enhanced rest facilities, improving the usability of electronic health records, and access to free parking and accommodation. Workload can be improved by reducing the size of outpatient clinics, which has benefits on surgeon and patient satisfaction. Insufficient staff numbers and an overload of administrative work can be addressed by employing allied health professionals such as physician associates to share the workload, as well as increasing their scope of practice. Furthermore, greater mentoring and support for junior doctors may help tackle the reducing numbers of doctors entering the profession.

The OLBI was used in this study to assess burnout. This is a validated tool for measuring burnout, with multiple studies supporting its use.^10-14^ It was developed in response to the Maslach Burnout Inventory, another commonly used tool, which did not include negatively worded questions.^33, 34^ The OLBI asks an equal mix of both positively and negatively worded questions to improve psychometric balancing.^10^ Furthermore, the Maslach Burnout Inventory assessed only the emotional component of exhaustion, whereas the OLBI aimed to also assess the physical and cognitive components of exhaustion.^10^ Convergence validity between the two tools has been demonstrated.^11,35^

### Study limitations

Limitations of this study were the number of respondents to the survey, which may have resulted in a self-selected group introducing possible selection and response bias. Furthermore, although respondents were from across all regions of the UK, a higher proportion were from London. Although to an extent this may be explained by higher population density, it is also in keeping with the base hospital of the primary authors, and thus may not be wholly representative of all orthopaedic surgeons across the country. Nevertheless, these findings highlight an important issue within the field and the modifiable work-related factors leading to burnout may help with healthcare resource planning.

## Conclusions

There was a moderate to high level of burnout among trauma and orthopaedic surgeons within the UK. Clinical and management teams should focus on improving staff numbers, reducing administrative work and increasing clinic consultation times to limit any further disengagement and exhaustion from surgeons. Reducing burnout among orthopaedic surgeons may help to improve mental wellbeing, work satisfaction and workforce productivity.

## Author Contributions

Conceptualisation: S.K.N. and K.V. Methodology: S.K.N. and F.A. Investigation: S.K.N. and F.A. Formal analysis: S.K.N. and B.K. Writing – original draft: S.K.N. and F.A. Writing – review and editing: S.K.N., F.A., B.K. and K.V. Supervision: K.V.
